# Construction of Spirooxindole Skeleton Through Intramolecular Dieckmann Cyclization

**DOI:** 10.1007/s13659-017-0131-0

**Published:** 2017-05-08

**Authors:** Ting Wu, Zhiqiang Pan, Chengfeng Xia

**Affiliations:** 1 The Key Laboratory of Chemistry for Natural Products of Guizhou Province, Chinese Academy of Sciences, Guiyang, 55002 China; 2 Key Laboratory of Medicinal Chemistry for Natural Resources, Ministry of Education, School of Chemical Science and Technology, Yunnan University, Kunming, 650091 China

**Keywords:** Dieckmann reaction, Cyclization, Spirooxindole, Indole

## Abstract

**Abstract:**

A highly efficient and direct approach was developed to construct the structurally diverse spirooxindole skeleton, which is an important basic motif in natural products. Both the 3,3′-pyrrolidonyl spirooxindoles and spiroindolin-2-one δ-lactones were smoothly obtained by the intramolecular Dieckmann cyclization of oxindoles in excellent yield under mild conditions.

**Graphical Abstract:**

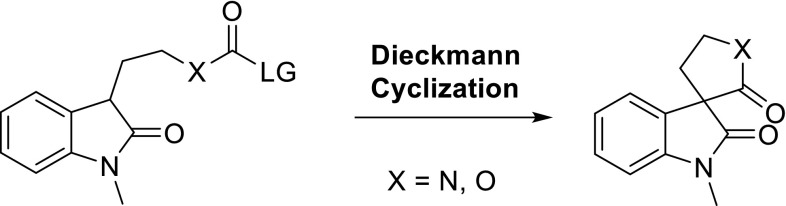

## Introduction

**Fig. 1:**
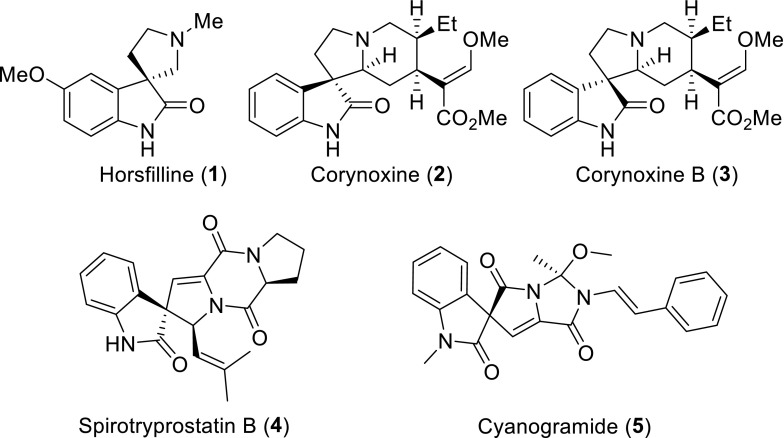
Representative natural products with spirooxindole skeleton

The spirooxindole ring system is a popular structure in a number of natural products and leading compounds, which possess significant biological and pharmaceutical activities (Fig. 1). Among them, horsfiline **1** was isolated from the leaves of the Malaysian indigenous tree *Horsfieldia superba* [1]. Corynoxine **2** and corynoxone B **3** are the important tetracyclic 3-spirooxindole alkaloids, which were isolated from the hooks of *Uncariae* plants, and have shown potential applications in the prevention or treatment of Parkinson’s disease [2–4]. Other complex spirooxindoles, such as spirotryprostatin B **4**, was produced by *Aspergillus fumigatu*s and found to inhibit mammalian cancer cell [5]. Cyanogramide **5** was derived from the fermentation broth of the marine-derived *Actinoalloteichus cyanogriseus* WH1-2216-6 and exhibited strong potencies to reverse the P-glycoprotein-mediated multidrug resistance [6].

**Scheme 1:**
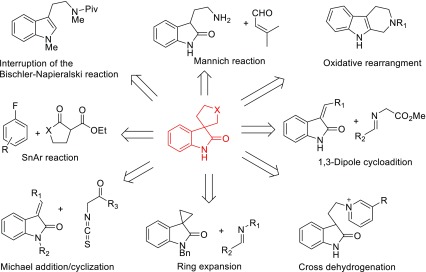
The general synthetic strategies of spirooxindoles

The important biological activities and structural complexities of spirooxindole alkaloids have attracted many organic chemists to develop a number of elegant synthetic strategies (Scheme 1). A representative application of the Mannich reaction in complex spirooxindole alkaloid synthesis was documented by Danishefsky in his approach to spirotryprostatin B **4** [7]. The biomimetically oxidative rearrangement of a tetrahydro-β-carboline was reported to construct the tetracyclic 3-spirooxindole skeleton by Martin [8]. The most widely used method for synthesis of structurally complex spirooxindole structure is the 1,3-dipolar cycloaddition (1,3-DC) reaction of azomethine ylide with olefin [9–17]. Moreover, Carreira et al. have developed a reliable methodology to access the pyrrolidinyl-spirooxindole structure via a MgI_2_-mediated ring-expansion reaction of a spiro[cyclopropane-1,3′′′oxindole] with an aldimine [18]. Other synthetic strategies of the spiro-pyrrolidone-3,3′-oxoindole frameworks also include Michael cyclization reaction [19–23] and C-selective SnAr reactions [24–26]. Besides, in our previous study, a completely different method has been achieved for the construction of the tetracyclic 3-spirooxindole through a transition-metal-free intramolecular cross-dehydrogenative coupling of pyridinium [27].

Although many elegant methods have been developed for the construction of spirooxindole skeletons, strategies that could directly and efficiently obtain the structurally diverse spirooxindoles are still limited. Therefore, searching for new approach for the construction of spirooxindole is still required. As a well-known organic reaction, Dieckmann cyclization was widely used in the preparation of natural products and medicines, but with limited reports on the construction of natural alkaloid skeletons [28–32]. In 2012, Zhang reported that bromoanilides underwent the Dieckmann condensation in the presence of copper (I) iodide and LHMDS to give the spirooxindoles [33]. In this work, we have developed a highly efficient and direct method to construct the spirooxindoles skeleton by the intramolecular Dieckmann cyclization.

## Results and Discussion

The starting 2-oxytryptamines **6** were easily prepared by direct oxidation of the corresponding tryptamine or tryptophol under modified conditions [34, 35]. At the outset, 2-oxytryptamine methoxyl carbamate **6a** was employed to screen the construction of spirooxindole by intramolecular Dieckmann cyclization. Unfortunately, no cyclized product was detected under various conditions (Table 1, entries 1–3). We speculated that the Dieckmann cyclization proceeded with the removal of methoxyl group during the reaction. However, the methoxyl group was a very weak leaving group, thus inhibited the cyclization process. To facilitate the cyclization, phenyl carbamate substrate **6b** was subjected to the reaction. As a result, the spirooxindole **7** was obtained in acceptable yield when KHMDS was used as base (Table 1, entry 4). Shift the leaving group from phenol to pentafluorophenol afforded the spirooxindole **7** excellent yield. Instead, when trifluoroethanol was used as leaving group, the generation of spirooxindole **7** was among the phenol and pentafluorophenol. We discovered that the best leaving group for the Dieckmann cyclization was *p*-nitrophenol and almost quantitative yield was achieved (Table 1, entry 7). Changing the base from KHMDS to NaHMDS or LHMDS also afforded excellent yields of cyclization (Table 1, entries 8 and 9). However, other bases, such as LDA, NaH, and *t*-BuOK, resulted in lower yields. When the weak bases (Et_3_N or K_2_CO_3_) were subjected, no spirooxindole product was generated (Table 1, entries 13 and 14).

**Table 1:** Optimization of Dieckmann cyclization

Entry	Substrate	LG	Base	Yield (%)^a^
1	**6a**	OMe	LHMDS	0
2	**6a**	OMe	NaH	0
3	**6a**	OMe	KHMDS	0
4	**6b**	OPh	KHMDS	63
5	**6c**	OC_6_F_5_	KHMDS	95
6	**6d**	OCH_2_CF_3_	KHMDS	83
7	**6e**	OPhNO_2_	KHMDS	Quant.
8	**6e**	OPhNO_2_	NaHMDS	Quant.
9	**6e**	OPhNO_2_	LHMDS	Quant.
10	**6e**	OPhNO_2_	LDA	73
11	**6e**	OPhNO_2_	NaH	85
12	**6e**	OPhNO_2_	*t*-BuOK	63
13	**6e**	OPhNO_2_	Et_3_N	0
14	**6e**	OPhNO_2_	K_2_CO_3_	0

^a^Isolated yields by silica gel column

With the established optimal conditions, different *p*-nitrophenol carbamates were conducted to investigate the scope of Dieckmann cyclization. It was found that the electron-withdrawing substitutions at C-5 position of tryptamine oxindoles gave lower yields (Table 2, compounds **10** and **11**). We reasoned that it might be due to the lower reactivities of the tryptamine oxindoles as nucleophile to attack the *p*-nitrophenol carbamates. When both of the N1 and N-terminal were substituted with different alkyl groups, the Dieckmann cyclization proceeded smoothly with good yields (Table 2, compounds **12**–**15**). Notably, when there was no protecting group at the N1 position of tryptamine oxindole, the cyclization also gave the target product in reasonable yield. This indicated that the C-3 hydrogen was earlier to be deprotonated than the hydrogen of N1. Besides the *p*-nitrophenol carbamate of tryptamine oxindoles, the *p*-nitrophenol carbonate of oxytryptophols were also examined for the Dieckmann cyclization. Although the yields were lower than carbamates, the corresponding spiroindolin-2-one *δ*-lactones **19** and **20** were obtained with acceptable yields.

**Table 2:** Substrate scope of the Dieckmann cyclization

In conclusion, we have developed a highly efficient and direct strategy for the construction of 3,3′-pyrrolidonyl spirooxindoles and spiroindolin-2-one *δ*-lactones under mild conditions. The reactions were achieved through an intramolecular Dieckmann cyclization of oxytrytamine carbamates to provide a series of spirooxindole derivatives in high yield.

## Experimental Section

### General Procedures for Synthesis of Spirooxindole

KHMDS (0.18 mmol) was added dropwise to a solution of indolin-2-one **6** (0.12 mmol) in anhydrous THF (5 mL) at 0 °C. After stirring for 30 min, the reaction temperature was allowed to warm to room temperature and continued stirring for additional 2 h. The reaction was quenched by saturated aqueous NH_4_Cl and extracted by CH_2_Cl_2_ (5 mL × 3). The combined organic layers was washed with brine, dried over anhydrous Na_2_SO_4_ and concerned. The residue was purified by silica gel column (CH_2_Cl_2_/acetone, 2:1) to afford the spirooxindole.

**7**. White powder (63% yield). ^1^H NMR (400 MHz, CDCl_3_) *δ*: 7.29 (t, *J* = 7.8 Hz, 1H), 7.14 (d, *J* = 7.5 Hz 1H), 7.06 (t, *J* = 7.5 Hz, 1H), 6.86 (d, *J* = 7.8 Hz, 1H), 3.78 (td, *J* = 8.8, 6.3 Hz, 1H), 3.59 (td, *J* = 9.0, 4.6 Hz, 1H), 3.21 (s, 3H), 2.97 (s, 3H), 2.67 (ddd, *J* = 13.0, 8.3, 4.6 Hz, 1H), 2.38 (ddd, *J* = 13.3, 8.5, 6.2 Hz, 1H); ^13^C NMR (100 MHz, CDCl_3_) *δ*: 175.7, 170.5, 144.5, 129.9, 129.0, 123.1, 122.8, 108.6, 57.8, 47.2, 30.5, 29.5, 26.6; HR–ESI–MS (m/z): calcd. for C_13_H_15_N_2_O_2_ [M + H]^+^, 231.1128, found 231.1130.

**8**. White powder (92% yield); ^1^H NMR (400 MHz, CDCl_3_) *δ*: 6.75 (dd, *J* = 8.4, 2.1 Hz, 1H), 6.69 –6.67 (m, 2H), 3.74–3.68 (m, 4H), 3.50 (td, *J* = 9.0, 4.5 Hz, 1H), 3.12 (s, 3H), 2.90 (s, 3H), 2.60 (ddd, *J* = 12.9, 8.2, 4.5 Hz, 1H), 2.45–2.18 (m, 1H). ^13^C NMR (100 MHz, CDCl_3_) *δ*: 174.3, 169.4, 155.3, 136.9, 130.0, 112.1, 109.5, 107.8, 57.1, 54.8, 46.1, 29.5, 28.5, 25.6; HR–ESI–MS (m/z): calcd. for C_13_H_15_N_2_O_2_ [M+H]^+^, 261.1234, found 261.1235.

**9**. White powder (89% yield); ^1^H NMR (400 MHz, CDCl_3_) *δ*: 7.09 (d, *J* = 7.9 Hz, 1H), 6.96 (s, 1H), 6.73 (d, *J* = 7.9 Hz, 1H), 3.76 (td, *J* = 8.7, 6.4 Hz, 1H), 3.57 (td, *J* = 9.0, 4.6 Hz, 1H), 3.18 (s, 3H), 2.96 (s, 3H), 2.65 (ddd, *J* = 13.0, 8.3, 4.6 Hz, 1H), 2.39–2.32 (m, 1H), 2.30 (s, 3H); ^13^C NMR (100 MHz, CDCl_3_) *δ*: 175.3, 170.4, 141.8, 132.3, 129.6, 128.9, 123.3, 107.9, 57.5, 46.9, 30.2, 29.2, 26.3, 20.8; HR–ESI–MS (m/z): calcd. for C_14_H_17_N_2_O_2_ [M+H]^+^, 245.1285, found 245.1286.

**10**. White powder (85% yield); ^1^H NMR (400 MHz, CDCl_3_) *δ*: 7.27–7.14 (m, 1H), 7.12 (d, *J* = 1.6 Hz, 1H), 6.77 (d, *J* = 8.3 Hz, 1H), 3.79 (dd, *J* = 15.4, 8.7 Hz, 1H), 3.57 (td, *J* = 9.1, 4.5 Hz, 1H), 3.19 (s, 3H), 2.97 (s, 3H), 2.67 (ddd, *J* = 13.0, 8.3, 4.5 Hz, 1H), 2.40–2.33 (m, 1H); ^13^C NMR (100 MHz, CDCl_3_) *δ*: 174.2, 168.8, 142.1, 130.4, 127.9, 127.3, 122.4, 108.4, 56.8, 46.1, 29.5, 28.3, 25.7; HR-ESI-MS (m/z): calcd. for C_13_H_14_ClN_2_O_2_ [M+H]^+^, 265.0738, found 265.0738.

**11**. White powder (81% yield); ^1^H NMR (400 MHz, CDCl_3_) *δ*: 7.08 (dd, *J* = 8.1, 5.2 Hz, 1H), 6.86–6.69 (m, 1H), 6.60 (dd, *J* = 8.8, 2.1 Hz, 1H), 3.84–3.72 (m, 1H), 3.56 (td, *J* = 9.1, 4.3 Hz, 1H), 3.19 (s, 3H), 2.97 (s, 3H), 2.65 (ddd, *J* = 12.8, 8.2, 4.3 Hz, 1H), 2.36 (ddd, *J* = 13.3, 8.6, 6.6 Hz, 1H); ^13^C NMR (100 MHz, CDCl_3_) *δ*: 174.9, 169.1, 162.5 (d, *J* = 245 Hz), 145.1 (d, *J* = 11 Hz), 123.5 (d, *J* = 118 Hz), 122.9, 108.0 (d, *J* = 23 Hz), 96.5 (d, *J* = 48 Hz), 56.3, 46.0, 29.5, 28.4, 25.7; HR–ESI–MS (m/z): calcd. for C_13_H_14_FN_2_O_2_ [M+H]^+^, 249.1034, found 249.1033.

**12**. White powder (70% yield); ^1^H NMR (400 MHz, CDCl_3_) *δ*: 7.15 (t, *J* = 7.8 Hz, 1H), 7.05 (d, *J* = 7.1 Hz, 1H), 6.95 (t, *J* = 7.5 Hz, 1H), 6.73 (d, *J* = 7.8 Hz, 1H), 5.85–5.60 (m, 1H), 5.14 (dd, *J* = 28.1, 13.8 Hz, 2H), 4.34–4.12 (m, 2H), 3.68 (td, *J* = 8.7, 6.4 Hz, 1H), 3.48 (td, *J* = 9.0, 4.6 Hz, 1H), 2.87 (s, 3H), 2.59 (ddd, *J* = 13.0, 8.3, 4.6 Hz, 1H), 2.30 (ddd, *J* = 13.3, 8.6, 6.3 Hz, 1H); ^13^C NMR (100 MHz, CDCl_3_) *δ*: 175.5, 170.4, 143.6, 130.7, 129.9, 128.8, 122.9, 122.8, 117.5, 109.3, 57.8, 47.2, 42.4, 30.5, 29.3; HR–ESI–MS (m/z): calcd. for C_15_H_17_N_2_O_2_ [M+H]^+^, 257.1285, found 257.1288.

**13**. White powder (80% yield); ^1^H NMR (400 MHz, CDCl_3_) *δ*: 7.31**–**7.10 (m, 5H), 7.10**–**6.98 (m, 2H), 6.92 (t, *J* = 7.5 Hz, 1H), 6.68**–**6.49 (m, 1H), 4.91 (d, *J* = 16.0 Hz, 1H), 4.73 (d, *J* = 16.0 Hz, 1H), 3.70 (dd, *J* = 15.1, 8.8 Hz, 1H), 3.51 (td, *J* = 9.0, 4.7 Hz, 1H), 2.88 (s, 3H), 2.64 (ddd, *J* = 13.1, 8.4, 4.7 Hz, 1H), 2.40**–**2.26 (m, 1H); ^13^C NMR (100 MHz, CDCl_3_) *δ*: 175.9, 170.5, 143.55, 135.3, 130.0, 128.9, 128.8, 127.5, 127.0, 123.0, 122.8, 109.5, 57.8, 47.2, 43.9, 30.5, 29.4; HR-ESI–MS (m/z): calcd. for C_19_H_19_N_2_O_2_ [M+H]^+^, 307.1441, found 307.1443.

**14**. White powder (60% yield); ^1^H NMR (400 MHz, CDCl_3_) *δ*: 7.31 (d, *J* = 10.3 Hz, 1H), 7.16 (d, *J* = 7.1 Hz, 1H), 7.07 (t, *J* = 7.5 Hz, 1H), 6.86 (d, *J* = 7.8 Hz, 1H), 5.80 (ddt, *J* = 16.2, 10.3, 5.9 Hz, 1H), 5.44–5.19 (m, 2H), 4.00 (dd, *J* = 3.8, 1.9 Hz, 2H), 3.84–3.67 (m, 1H), 3.55 (td, *J* = 9.0, 4.6 Hz, 1H), 3.22 (s, 3H), 2.68 (ddd, *J* = 12.9, 8.2, 4.5 Hz, 1H), 2.39 (ddd, *J* = 13.2, 8.5, 6.4 Hz, 1H). ^13^C NMR (100 MHz, CDCl_3_) *δ*: 175.6, 170.3, 144.5, 131.9, 129.8, 129.0, 123.0, 122.7, 118.3, 108.5, 58.0, 46.0, 44.6, 29.5, 26.5; HR–ESI–MS (m/z): calcd. for C1_5_H_17_N_2_O_2_ [M+H]^+^, 257.1285, found 257.1286.

**15**. White powder (75% yield); ^1^H NMR (400 MHz, CDCl_3_) *δ*: 7.41–7.27 (m, 6H), 7.18–7.03 (m, 2H), 6.87 (d, *J* = 7.8 Hz, 1H), 4.64 (d, *J* = 16.0 Hz, 1H), 4.50 (d, *J* = 16.0 Hz, 1H), 3.67 (td, *J* = 8.9, 6.1 Hz, 1H), 3.44 (td, *J* = 9.0, 4.9 Hz, 1H), 3.24 (s, 3H), 2.66 (ddd, *J* = 13.2, 8.4, 4.9 Hz, 1H), 2.56–2.24 (m, 1H); ^13^C NMR (100 MHz, CDCl_3_) *δ*: 175.5, 170.68, 144.5, 135.9, 129.9, 129.1, 128.8, 128.1, 127.7, 123.1, 122.7, 108.6, 57.9, 47.4, 44.5, 29.4, 26.6; HR-ESI–MS (m/z): calcd. for C_19_H_19_N2O_2_ [M + H]^+^, 307.1441, 307.1442.

**16**. White powder (43% yield); ^1^H NMR (400 MHz, CDCl_3_) *δ*: 8.17 (brs, 1H), 7.20 (t, *J* = 7.7 Hz, 1H), 7.13 (d, *J* = 7.3 Hz, 1H), 7.02 (t, *J* = 7.5 Hz, 1H), 6.84 (d, *J* = 7.8 Hz, 1H), 3.77 (td, *J* = 8.9, 6.1 Hz, 1H), 3.60 (td, *J* = 9.0, 4.8 Hz, 1H), 2.99 (s, 3H), 2.72 (ddd, *J* = 13.2, 8.4, 4.8 Hz, 1H), 2.56–2.29 (m, 1H); ^13^C NMR (100 MHz, CDCl_3_) *δ*: 177.3, 170.5, 141.6, 130.4, 128.9, 123.1, 122.9, 110.2, 58.2, 47.3, 30.6, 29.4; HR–ESI–MS (m/z): calcd. for C_12_H_12_N_2_O_2_ [M+H]^+^, 217.0972, found 217.0974.

**17**. White powder (58% yield); ^1^H NMR (400 MHz, CDCl_3_) *δ*: 7.37 (t, *J* = 7.7 Hz, 1H), 7.23 (d, *J* = 7.3 Hz, 1H), 7.12 (t, *J* = 7.5 Hz, 1H), 6.90 (d, *J* = 7.8 Hz, 1H), 4.62 (td, *J* = 8.5, 5.0 Hz, 1H), 4.62 (td, *J* = 8.5, 5.0 Hz, 1H), 3.25 (s, 3H), 2.91 (ddd, *J* = 12.7, 7.4, 5.0 Hz, 1H), 2.67 (dt, *J* = 13.2, 7.9 Hz, 1H); ^13^C NMR (100 MHz, CDCl_3_) *δ*: 173.3, 172.9, 144.4, 129.7, 127.7, 123.5, 123.0, 108.9, 66.7, 55.6, 33.5, 26.7; HR–ESI–MS (m/z): calcd. for C_12_H_12_NO_3_ [M+H]^+^, 218.0812, found 218.0809.

**18**. White powder (30% yield); ^1^H NMR (400 MHz, CDCl_3_) *δ*: 7.49 (dd, *J* = 8.3, 1.6 Hz, 1H), 7.34 (d, *J* = 1.5 Hz, 1H), 6.77 (d, *J* = 8.3 Hz, 1H), 4.81 (q, *J* = 7.9 Hz, 1H), 4.60 (td, *J* = 8.6, 4.7 Hz, 1H), 3.23 (d, *J* = 8.3 Hz, 3H), 2.89 (ddd, *J* = 12.5, 7.5, 4.7 Hz, 1H), 2.66 (dt, *J* = 13.2, 8.1 Hz, 1H); ^13^C NMR (100 MHz, CDCl_3_) *δ*: 172.7, 172.2, 143.5, 132.6, 129.5, 126.4, 115.9, 110.3, 66.8, 55.6, 33.4, 26.8; HR–ESI–MS (m/z): calcd. for C_12_H_10_NBrO_3_K [M+K]^+^, 333.9476, found 333.9484.

**19**. White powder (43% yield); ^1^H NMR (400 MHz, CDCl_3_) *δ*: 6.88 (dd, *J* = 8.4, 2.2 Hz, 1H), 6.83 (d, *J* = 2.1 Hz, 1H), 6.80 (d, *J* = 8.5 Hz, 1H), 4.82 (q, *J* = 7.9 Hz, 1H), 4.60 (td, *J* = 8.5, 5.0 Hz, 1H), 3.79 (s, 3H), 3.22 (s, 3H), 2.90 (ddd, *J* = 12.5, 7.5, 4.7 Hz, 1H), 2.66 (dt, *J* = 13.2, 8.1 Hz, 1H); ^13^C NMR (100 MHz, CDCl_3_) *δ*: 172.9, 172.9, 156.6, 137.8, 128.8, 113.9, 110.6, 109.3, 66.7, 55.9, 55.9, 33.6, 26.8; HR–ESI–MS (m/z): calcd. for C_13_H_14_NO_4_ [M+H]^+^, 248.0917, found 248.0916.
